# Assessment of Mycotoxin Exposure in Breastfeeding Mothers with Celiac Disease

**DOI:** 10.3390/nu10030336

**Published:** 2018-03-10

**Authors:** Francesco Valitutti, Barbara De Santis, Chiara Maria Trovato, Monica Montuori, Simona Gatti, Salvatore Oliva, Carlo Brera, Carlo Catassi

**Affiliations:** 1 Pediatric Gastroenterology and Liver Unit, Sapienza University of Rome, 00161 Rome, Italy; francesco.valitutti@uniroma1.it (F.V.); chiaramaria.trovato@uniroma1.it (C.M.T.); monica.montuori@uniroma1.it (M.M.); salvatore.oliva@uniroma1.it (S.O.); 2 Laboratory for Mycotoxins, Istituto Superiore di Sanità, 00161 Rome, Italy; barbara.desantis@iss.it (B.D.S.); carlo.brera@iss.it (C.B.); 3 Department of Pediatrics, Università Politecnica delle Marche, 60123 Ancona, Italy; simona.gatti@hotmail.it

**Keywords:** celiac disease, mycotoxins, breast milk

## Abstract

Objective: To assess the risk of mycotoxin exposure (aflatoxin M1, ochratoxin A, and zearalenone) in celiac disease (CD) breastfeeding mothers and healthy control mothers, as well as in their offspring, by quantifying these contaminants in breast milk. Study design: Thirty-five breastfeeding women with CD on a gluten-free diet and 30 healthy breastfeeding controls were recruited. Milk sampling was performed three times per day for three consecutive days. Mycotoxin content was investigated by an analytical method using immunoaffinity column clean-up and high-performance liquid chromatography (HPLC) with fluorometric detection. Results: Aflatoxin M1 (AFM1) was detected in 37% of CD group samples (mean ± SD = 0.012 ± 0.011 ng/mL; range = 0.003–0.340 ng/mL). The control group showed lower mean AFM1 concentration levels in 24% of the analyzed samples (0.009 ± 0.007 ng/mL; range = 0.003–0.067 ng/mL, ANOVA on ranks, *p*-value < 0.01). Ochratoxin A and zearalenone did not differ in both groups. Conclusion: Breast milk AFM1 contamination for both groups is lower than the European safety threshold. However, the estimated exposures of infants from CD mothers and control mothers was much higher (≃15 times and ≃11 times, respectively) than the threshold set by the joint FAO/WHO Expert Committee on Food Additives (JECFA). Since incongruities exist between JECFA and the European Union standard, a novel regulatory review of the available data on this topic is desirable. Protecting babies from a neglected risk of high AFM1 exposure requires prompt regulatory and food-control policies.

## 1. Introduction

Mycotoxins are low molecular weight secondary metabolites produced by several fungi genera such as Aspergillus, Fusarium, and Penicillium, which represent an important health concern because of their toxic effects such as genotoxicity, carcinogenicity, teratogenicity, and immunosuppression [1,2]. Under particular environmental conditions (i.e., temperature and humidity) and/or biotic stress, these fungi may grow on crops and food commodities, as well as during storage, and activate the metabolism that leads to mycotoxin production [3]. The most relevant mycotoxins are aflatoxin B1 (AFB1), ochratoxin A (OTA), and fusarium toxins, e.g., zearalenone (ZEA). Frequently found in the milk of exposed lactating mammals, aflatoxin M1 (AFM1) is a product of detoxification of AFB1 and is considered by the International Agency for Research on Cancer (IARC) as possibly carcinogenic to humans [4]. OTA is carcinogenic in rodents and nephrotoxic in many mammal species; it has been also classified by IARC as possibly carcinogenic to humans and its presence in breast milk has been widely outlined [5]. The biomonitoring of ZEA is also important due to its toxic effects mainly on the reproductive system [6]; moreover, ZEA contamination was previously described in breast milk in Italy [7]. Consumers can be at risk of mycotoxins exposure both directly, by consuming contaminated grains, seeds, and dried fruits, or indirectly, by consuming contaminated animal source food (e.g., milk) derived from animals exposed to these contaminants. Foods at risk of mycotoxin contaminations are mainly cereals, accounting for 50% intake, followed by dried fruits, spices, dairy products, alcoholic beverages, and coffee [8]. Among cereals, corn is largely subject to some xenobiotic contamination and may present the potential co-occurrence of more than one mycotoxin [9]. The high consumption of corn can be of great concern, not only in the case of particular local diet habits, but also for specific disease-related dietary regimens. CD is an immune-mediated systemic disorder elicited in genetically susceptible individuals by gluten, which is a protein found in wheat and some related cereals [10]. Gluten-free diet (GFD) is the only treatment for celiac disease (CD) [11]. GFD is characterized by a higher consumption of corn, rice, and other gluten-free cereals. In the last decade, some reports have been focused on corn-based products, in some cases showing heavy mycotoxin contamination [12,13]. More recently, specific surveys on the mycotoxin contamination of GFD products have been published with conflicting results [14,15,16,17]. However, whilst breast milk levels of AFM1 have been detected worldwide in exposure assessment studies, so far no study has evaluated the exposure to mycotoxins in breast milk samples taken from patients with CD on treatment with the GFD.

The aim of our study was to assess the risk of mycotoxin exposure (namely aflatoxin M1, ochratoxin A, and zearalenone) in CD mothers and healthy control mothers, by quantifying these contaminants in breast milk. The other fusarium toxins which can be present in corn (namely fumonisins and deoxynivalenol) were not analyzed, for both analytical reasons (the method was set for HPLC-FL without derivatization) and because they are hardly secreted in breast milk [18]. A nutritional survey specifically aimed at quantifying corn consumption both in CD breastfeeding mothers and controls was undertaken. Other cereal intake was not measured in the study survey. 

## 2. Materials and Methods 

During the study period of 2011–2013, 35 breastfeeding women with CD on treatment with the GFD for at least 12 months and 30 healthy breastfeeding controls were recruited at Umberto I University Hospital in Rome, and Salesi Children’s Hospital in Ancona, or through a specific study call run by the Italian Celiac Society on social media (celiac society national magazine, website, Facebook, Twitter, newsletters). All recruited women with CD completed the study, while only 23 out of 30 control women collected breast milk samples for study purpose.

All women with CD had been previously diagnosed by duodenal biopsy at secondary/tertiary care facilities. 

Under the approval and jurisdiction of the Ancona Ethical Committee, an informed consent was signed by participants. The project was carried out in accordance with The Code of Ethics of the World Medical Association (Declaration of Helsinki).

### 2.1. Exclusion Criteria

(A) Lactation in the first two weeks after delivery for the following reasons: to avoid the generation of anxiety in breastfeeding women at-risk for stress-induced hypogalactia in the early weeks of lactation; and to consider only mature milk rather than colostrum in the analysis.
(B) Lactation for more than four months, as we attempted to estimate exposure to mycotoxins in breastfed babies rather than in children on complementary feeding during weaning.
(C) Diagnosis of diabetes mellitus (both type-1 and type-2), since it might have slightly changed food choices.
(D) Feeding disorders or food allergy, since it might have slightly changed food choices.
(E) Being on a restricted diet for medical/ethical/religious reasons (including non-celiac gluten sensitivity among controls), since it might have slightly changed food choices.
(F) Family history of CD (control group only). This exclusion criterion was set to minimize possible switch to gluten-free foods by chance (e.g., shared meals in family) during the study period.

### 2.2. Human Milk Analysis 

Human milk was collected at home and promptly frozen at −18 °C. Milk sampling was performed three times per day throughout three days, as follows: the first milk sample was collected in the morning during fasting, the second 4 h after lunch, and the third 2 h after dinner. Nine samples were obtained from each mother. This sampling schedule aimed to identify possible patterns of mycotoxin contamination: baseline (fasting on day 1); absorption; metabolism; breast milk secretion; specific associations with food. Frozen samples were then delivered to the Unit of “Genetically Modified Organisms and Mycotoxins” at the Italian National Institute of Health, Rome. A constant temperature of −18 °C was guaranteed during delivery. With the exception of insufficient samples (<5 mL) for analyses (*n* = 40 for CD mothers and *n* = 29 for control mothers), mycotoxin content in breast milk was investigated by an analytical method using immunoaffinity column clean-up and high-performance liquid chromatography (HPLC) with fluorometric detection. The method was in-house validated with performance complying with legislation requirements (Regulation 401/2006) and reached the following limit of quantification (LOQ): 0.007 ng/mL for AFM1, 0.034 ng/mL for OTA, and 4.0 ng/mL for ZEA. In order to handle the concentration data below the limit of detection, a left-censored data was managed by a substitution method following a middle bound approach; thus, for results below the LOQ, a numerical value equal to half of the LOQ, namely 0.0035 ng/mL for aflatoxin M1, 2.0 ng/mL for zearalenone, and 0.017 ng/mL for ochratoxin A, was used.

### 2.3. Dietary Survey 

To quantify the dietary intake cereal-based products, both CD and control subjects were asked to record their cereal consumption during the three days of breast milk collection by food diaries. During the days of the survey, participants were advised to maintain their usual eating habits. Breastfeeding mothers registered all cereal-based products consumed in detail (sweet pastries, bread, pasta, pizza, homemade cakes, biscuits, crackers, breakfast cereals, breadcrumb, and other cereals), specifying the type, brand, and weight by means of a digital scale. For each “out of home” meal, instead of weighing they used a photo atlas to determine the weight of food consumed (photo atlas of food portions, Istituto Scotti Bassani 2008). “Out of home” meals accounted only for 2.4% of all recorded meals. 

Corn intake (mean and standard deviation) was estimated for each participant based on the three-day diaries. Dietary records enabled us to calculate corn intake based on the specific brand/type of products consumed during the survey. Eighty-nine percent of the recorded meals were fully detailed as regards brand/type/weight/percentage of corn in the ingredient list. Eleven percent of the recorded meals were either registered approximatively by photo atlas reference or—due to the lack of corn percentage in the ingredient list—the hypothetical corn content was estimated as the mean corn content of similar products present on the Italian gluten-free market. 

### 2.4. Statistics 

The Mann-Whitney test was used to compare group consumption data as appropriate. Linear regression analysis (Pearson χ2 test) was applied to assess a possible correlation between daily corn intake and breast milk mycotoxin concentration. Differences between concentration levels of mycotoxins in the CD and control groups were explored by applying Kruskal-Wallis, Wilcoxon signed-rank, and Wilcoxon rank-sum tests. Statistical analysis was performed by Stata/IC 14.0, Copyright 1985-2015 StataCorp, and by SPSS version 17.0 software (SPSS Inc., Chicago, IL, USA).

## 3. Results

Demographic data are summarized in Table 1. Neither groups showed any significant difference for each of the demographics considered.

Data obtained from the analysis of breast milk samples in both groups (CD and control mothers) are summarized in Table 2.

Overall, 453 samples were suitable for analysis: 275 from 35 CD mothers and 178 from 23 control mothers (seven drop-outs among controls); 40 samples from CD mothers and 29 samples from controls were insufficient (<5 mL) for analysis purpose. 

AFM1 was detected in 37% of CD group samples (mean ± SD = 0.012 ± 0.011 ng/mL; range = 0.003–0.340 ng/mL). A slightly higher mean concentration of AFM1 was found in those samples collected during fasting (0.017 ± 0.028 ng/mL), which showed a statistically significant difference when compared to those collected 4 h after lunch and 2 h after dinner (0.011 ± 0.010 ng/mL and 0.009 ± 0.006 ng/mL, respectively, ANOVA on ranks, *p*-value < 0.001). When compared to mothers with CD, the control group showed lower mean AFM1 concentration level in 24% of the analyzed samples (0.009 ± 0.007 ng/mL; range = 0.003–0.067 ng/mL, ANOVA on ranks, *p*-value < 0.01).

**Figure 1:**
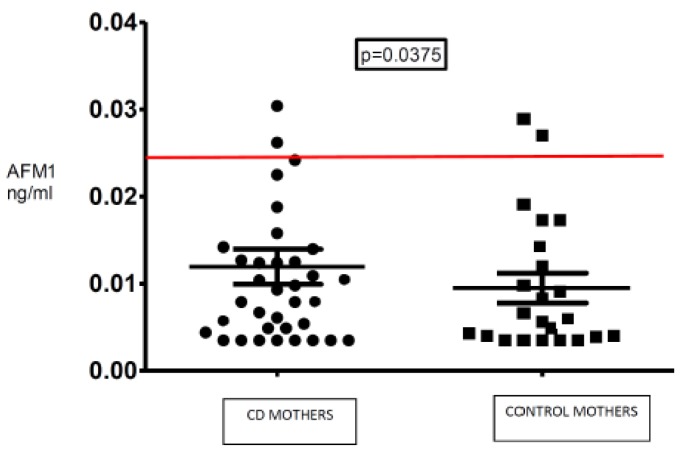
Aflatoxin M1 (AFM1) mean breast milk concentration for each of the CD and control mothers. Means were obtained by averaging individual samples for each subject. The red line represents the maximum AFM1 threshold established by the European Union for infant formulas.

Comparison between AFM1 mean breast milk concentration for each CD and control mother is shown in Figure 1. 

In both groups, AFM1 maximum levels were registered on the first day of collection, during the fasting period (0.34 ng/mL and 0.067 ng/mL for CD mothers and control mothers, respectively).

ZEA was detected in 4% of CD mothers’ milk samples (mean ± SD = 2.17 ± 0.41 ng/mL; range = 2.0–17.0 ng/mL) and in 8% of the control group samples (mean ± SD = 2.76 ± 1.77 ng/mL; range = 2.5–21.9 ng/mL). The comparison between levels of ZEA in breastfeeding women with CD and in healthy breastfeeding controls did not show any statistical difference. 

Ochratoxin A was found in six samples from mothers with CD: one single sample from one mother and five samples from another mother were positive for this mycotoxin; on the contrary, it was present only in one single sample from the control group.

Mean corn daily intake was 104.6 g (SD ± 49.1 g; range: 0–244.1 g) in the CD breastfeeding group, which was significantly higher (*p*-value ˂ 0.01, Student’s *t*-test) compared to the breastfeeding control group (mean: 6.23 g; SD ± 14.9 g; range: 0–76 g). 

**Figure 2:**
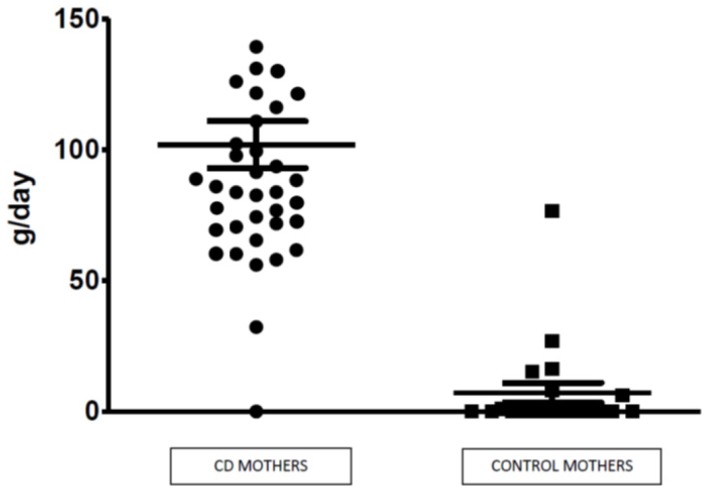
Daily corn intake of CD breastfeeding mothers and control mothers.

Corn consumption in the two groups is shown in Figure 2.

As regards the consumption of different cereal-based products, no differences were observed comparing the whole CD breastfeeding group to the breastfeeding controls. Linear regression analysis did not show any correlation between corn consumption and the three mycotoxins analyzed in both groups.

## 4. Discussion

Mycotoxins represent a food hazard of great concern, both in developed and developing countries. The European Union has established the maximum level of mycotoxins for a broad number of foods and food products (cereals, nuts, dried fruits, cocoa, and milk, among others). More precisely, a limit of 0.025 µg/kg has been set for AFM1 levels in infant formulas and follow-on formulas. On the other hand, the evaluation of the joint FAO/WHO Expert Committee on Food Additives (JECFA) established in 2002 that the intake of AFM1 should be lower than 0.11 ng/kg bw/day, based on proposed maximum levels of AFM1 contamination in cow’s milk of 0.023 µg/kg for the GEMS/Food European-type diet [19].

Corn-based diet is at higher risk for mycotoxin overload, namely AFM1 and ZEA [16,20,21], representing a possible concern for patients with CD who must strictly comply with a gluten-free diet. 

Although considerable progress has been made regarding CD epidemiology, pathogenesis, and diagnosis, a strict gluten-free diet is still the only of treatment for this disorder, since the first description made by the Dutch physician Dicke in the early 1950s [22]. GFD entails avoidance of gluten-containing grains such as wheat, rye, and barley, whereas acceptable grains include rice, oats, buckwheat, corn, millet, and quinoa [23].

The studies conducted worldwide (e.g., Brazil, Cameroon, Egypt, Iran, and other countries) on the presence of mycotoxins in breast milk from non-CD mothers demonstrate that monitoring this biomarker is important for the exposure assessment studies of both adults and newborns [24,25,26,27,28,29,30].

In the present study, we aimed to assess whether the diet habits of this special population with CD lead to different exposure trends. To the best of our knowledge, this is the first study assessing mycotoxin exposure (namely AFM1, OTA, and ZEA) on biologic samples in patients with CD and the subsequent risk of exposure in a breastfed cohort of children. 

An infant needs roughly 107 kcals/kg/day for the first three months of life due to their extremely fast growth [31]. One hundred milliliters of mature breast milk contain 70 kilocalories. In order to fulfil their energy requirement, the daily milk consumption of babies aged 0–3 months and weighing 3.6 kg to 6.0 kg ranges approximately from 525 mL to 875 mL [32,33]. Therefore, according to these caloric needs and our average weight data for both cohorts, babies from our CD mothers and control mothers would theoretically drink 700 g of breast milk per day. Based on the estimated daily average milk consumption of 700 g in babies from CD mothers and babies from controls according to their mean weights, respectively, the baby AFM1 exposure from mothers with CD was 1.6 ng/kg bw/day, while it was 1.2 ng/kg bw/day from control mothers. The exposure of newborns from CD mothers was thus much higher (≃15 higher times) than the threshold set by the joint FAO/WHO Expert Committee on Food Additives (JECFA) [19]. However, according to our data from control mothers, the general scenario is far from reassuring: their exposure is also above JECFA threshold (≃11 times higher).

The EU regulation established a maximum AFM1 content of 0.025 µg/kg (i.e., 0.025 ng/mL, approximating milk density to 1 g/mL) for infant formulas and follow-up milk [34]. Previous data on infant formula analysis from non-EU and EU countries have shown that mycotoxin content is generally very low and in the vast majority of cases it does not reach the threshold set by the European commission [35,36,37,38,39].

The average values of our results on breastfed children fall within the European safety threshold. However, since discrepancies exist with other standards, such as the JECFA threshold, whether EU regulation really puts our children on the safest side remains to be addressed. 

With regard to ZEA intake, the resulting contamination and the number of values > LOQ were low; when considering average body weight and average consumption values, an exposure value lower than the ZEA tolerable daily intake (TDI) of 1 μg/kg of body weight was obtained. 

Only two CD mothers and one control mother showed the presence of Ochratoxin A. These data are definitely reassuring, albeit they are in contrast with a previous study conducted in Italy on breast milk which revealed that 70% of the samples from healthy women were positive for Ochratoxin A (*n* = 82, range 0.005 ng/mL–0.405 ng/mL) [40].

In the present study, the other fusarium toxins which can contaminate corn (namely fumonisins and deoxynivalenol) were not analyzed, both for analytical reasons (the method was set for HPLC-FL without any derivatization step) and because they are hardly secreted in human milk [18].

We are aware of some limitations of our study. The restricted nutritional survey (cereal-based) might have prevented us from identifying other possible foods which also undergo mycotoxin contamination. A duplicate diet, with analysis on both food as well breast milk samples, would have been more precise but extremely less feasible. Moreover, being in a study including a nutritional survey might have biased behaviors and food choices of participants, particularly CD women who were more aware about the study rationale. A higher number of recruited subjects and a higher compliance rate with breast milk collection would have allowed us to better circumstantiate our findings which, ultimately, describe a non-frequent event. 

Nevertheless, the strength of our study is that the information is directly related to the mycotoxin intake of the baby when breastfed. Although mothers’ exposure was considered safe throughout the study, concerns could arise with regard to newborn/baby diet, mainly in children from CD women who showed higher exposure risk. 

## 5. Conclusions

Our data pinpoint the importance of improving food safety about mycotoxin contamination. Since incongruities exist between JECFA and EU standards, we hope that our work could trigger a novel regulatory review of the available data on this topic. 

Protecting babies from a neglected risk of high AFM1 exposure requires prompt regulatory and food-control policies. For the sake of particularly vulnerable infants breastfed by CD mothers, as well all breastfed infants, this should be considered a matter of priority.

## Figures and Tables

**Table 1:** Demographic data at sampling time for both mothers’ groups are represented as mean (±standard deviation).

	Celiac Disease (CD) Mothers	Control Mothers
Maternal age	31.6 years (±6.4 years)	33.1 years (±8.5 years)
Maternal weight	66.1 kg (±11.4 kg)	61.7 kg (±16.5 kg)
Children age	1.5 months (±0.8 months)	1.9 months (±1.0 months)
Children weight	4.8 kg (±1.3 kg)	5.3 kg (±0.7 kg)

**Table 2:** Presence of the three studied mycotoxins from both groups. Overall, 453 samples were suitable for analysis: 275 from CD mothers and 178 from control mothers.

	AFM1	ZEA	OTA
**CD mothers—*n* of sample positives (%)**	104 (37%)	12 (4%)	6 (2%)
Mean (ng/mL)	0.012	2.1	NA
Median (ng/mL)	0.010	2.9	NA
Range (ng/mL)	0.003–0.340	2.0–17	0.017–0.123
**Controls—*n* of sample positives (%)**	43 (24%)	15 (8%)	1 (0.5%)
Mean (ng/mL)	0.009	2.7	NA
Median (ng/mL)	0.005	2.2	NA
Range (ng/mL)	0.003–0.067	2.0–22	0.017–0.056

AFM1: Aflatoxin M1; ZEA: Zearalenone; OTA: Ochratoxin A.
